# Rhinoscleroma pathogenesis: The type K3 capsule of *Klebsiella rhinoscleromatis* is a virulence factor not involved in Mikulicz cells formation

**DOI:** 10.1371/journal.pntd.0006201

**Published:** 2018-01-30

**Authors:** Barbara Corelli, Ana S. Almeida, Fabiane Sonego, Virginia Castiglia, Cindy Fevre, Sylvain Brisse, Philippe J. Sansonetti, Régis Tournebize

**Affiliations:** 1 Molecular Microbial Pathogenesis Unit, Department of Cell Biology and Infection, Institut Pasteur, Paris, France; 2 INSERM U1202, Paris, France; 3 Unit of Technology and Service Photonic BioImaging (UTechS PBI), Center for Innovation and Technological Research (Citech), Institut Pasteur, Paris, France; 4 Université Paris Diderot, Sorbonne Paris Cité, Paris, France; 5 Genotyping of Pathogens and Public Health, Institut Pasteur, Paris, France; 6 Biodiversity and Epidemiology of Bacterial Pathogens, Institut Pasteur, Paris, France; 7 Chaire de Microbiologie et Maladies Infectieuses, Collège de France, Paris, France; University of California San Diego School of Medicine, UNITED STATES

## Abstract

Rhinoscleroma is a human specific chronic granulomatous infection of the nose and upper airways caused by the Gram-negative bacterium *Klebsiella pneumoniae* subsp. *rhinoscleromatis*. Although considered a rare disease, it is endemic in low-income countries where hygienic conditions are poor. A hallmark of this pathology is the appearance of atypical foamy monocytes called Mikulicz cells. However, the pathogenesis of rhinoscleroma remains poorly investigated. Capsule polysaccharide (CPS) is a prominent virulence factor in bacteria. All *K*. *rhinoscleromatis* strains are of K3 serotype, suggesting that CPS can be an important driver of rhinoscleroma disease. In this study, we describe the creation of the first mutant of *K*. *rhinoscleromatis*, inactivated in its capsule export machinery. Using a murine model recapitulating the formation of Mikulicz cells in lungs, we observed that a *K*. *rhinoscleromatis* CPS mutant (KR *cps*^*-*^) is strongly attenuated and that mice infected with a high dose of KR *cps*^*-*^ are still able to induce Mikulicz cells formation, unlike a *K*. *pneumoniae* capsule mutant, and to partially recapitulate the characteristic strong production of IL-10. Altogether, the results of this study show that CPS is a virulence factor of *K*. *rhinoscleromatis* not involved in the specific appearance of Mikulicz cells.

## Author summary

Rhinoscleroma is a human specific chronic infection characterized by the formation of granuloma in the nose and upper airways. It is a rare disease endemic in low-income countries where hygienic conditions are poor and caused by the Gram-negative bacterium *Klebsiella pneumoniae* subsp. *rhinoscleromatis*. A hallmark of this pathology is the appearance of atypical foamy monocytes called Mikulicz cells. Very little is known about the cellular and molecular mechanisms underlying this disease and the bacterial virulence factors of *K*. *rhinoscleromatis* are unknown. In this study, we created the first mutant made in *K*. *rhinoscleromatis* and inactivated the production of capsule, an outer-membrane-anchored polysaccharide. Using a murine model recapitulating the formation of Mikulicz cells and this bacterial capsule mutant, we observed that capsule is a virulence factor for *K*. *rhinoscleromatis* which is not required for the formation of Mikulicz cells, indicating that other specific virulence factors are present in the genome of the bacterium. This works opens the way to further genetic analysis of *K*. *rhinoscleromatis* and identification of new specific virulence factors.

## Introduction

Rhinoscleroma is a chronic granulomatous infectious disease that affects the nose and other parts of the respiratory tract down to the trachea [1]. Although few sporadic cases are typically described in Western Europe and in the USA, this disease is still endemic in impoverished areas of the Middle East, Eastern Europe, tropical Africa, South East Asia, Central and South America. A delay in the diagnosis can lead to complications such as physical deformity, upper airway obstruction and, rarely, sepsis. Treatment can be challenging and includes surgery and prolonged course of antibiotics to avoid relapses. The bacterium implicated as the causative agent of rhinoscleroma is *Klebsiella pneumoniae* subsp. *rhinoscleromatis* (hereafter mentioned as *K*. *rhinoscleromatis* or KR), a subspecies of *Klebsiella pneumoniae*. Despite being geographically broadly distributed, *K*. *rhinoscleromatis* has been isolated mainly in human [2] although three recent reports mention the identification of *K*. *rhinoscleromatis* in cockroaches [3,4] or chickens [5] in low hygiene settings. *K*. *rhinoscleromatis* is very closely related to *Klebsiella pneumoniae* subsp. *pneumoniae* but can be distinguished from *K*. *pneumoniae sensu stricto* by biochemical properties and multilocus sequence typing [6].

Rhinoscleroma development is typically described clinically and pathologically into three overlapping stages: catarrhal stage, proliferative stage, and sclerotic stage [7]. The catarrhal stage is marked by purulent rhinorroea and nasal obstruction, which persists for months. Histological examination shows evidence of squamous metaplasia with a subepithelial infiltrate of polymorphonuclear cells. However, in the subepithelial layer, bacteria are incompletely digested and further released into tissues. The proliferative stage is characterized by symptoms of epistaxis, nasal deformity and other problems depending on the other areas affected. In addition, histology shows the appearance of Mikulicz cells, a hallmark of rhinoscleroma [8]. These cells are large foamy macrophages with numerous enlarged vacuoles containing viable or non-viable bacteria. Finally, the sclerotic stage is characterized by increasing deformity, granulomatous areas and scar formation. Most patients are diagnosed in the proliferative stage, when the lesion appears as a bluish-red, rubbery granuloma and the typical Mikulicz cells can be observed.

Mikulicz cells are only documented in rhinoscleroma and have been described as atypical inflammatory monocytes specifically recruited from the bone-marrow upon *K*. *rhinoscleromatis* infection [9]. These cells represent a peculiar state of highly vacuolated inflammatory monocytes unable to digest bacteria. Moreover, it has been shown that IL-10, an anti-inflammatory cytokine, is essential in the establishment of a proper environment leading to the phenotypic maturation of Mikulicz cells [9].

Different virulence factors have been implicated in the pathogenesis of *K*. *pneumoniae*. Capsule polysaccharide (CPS) is recognized as one of the most important virulence determinants of this pathogen. The presence of CPS inhibits the deposition of complement components onto the bacterium [10–12], impedes adhesion and reduces phagocytosis of the bacterium by macrophages and epithelial cells [10,12–17]. Using *in vivo* models of colonization and pathogenesis, CPS mutants have been shown to be unable to colonize either pulmonary or systemic tissues [13,18,19]. Clearly, CPS plays an important role in the interplay between *K*. *pneumoniae* and the innate immune system.

*K*. *pneumoniae* and *K*. *rhinoscleromatis* are heavily capsulated bacteria. *K*. *pneumoniae* express 134 different capsular serotypes that they are easily transferred via homologous recombination [20,21]. Interestingly, despite their scattered geographical distribution, all *K*. *rhinoscleromatis* isolates belong to capsular type 3 (K3) [6]. This is raising the question of whether the K3 serotype capsule composition plays any specific role in rhinoscleroma pathology. Indeed the K3 capsule repeated unit is rich in mannose residues, its repeated unit being composed of →2-[(4,6-(*S*)-pyruvate)-α-D-Man-(1→4)]-α-D-GalA-(1→3)-α-D-Man-(1→2)-α-D-Man-(1→3)-ß-D-Gal-(1→ [22]. This is also suggestive of possible interaction of the bacteria with mannose receptors mainly carried by macrophages and dendritic cells. Indeed, the K3 capsule has been shown to be one of the few *Klebsiella* K types able to bind to the mannose receptor [23]. The complete sequence of the genomic region comprising the capsule polysaccharide synthesis gene cluster was determined [24]. However, to date, the link between CPS and *K*. *rhinoscleromatis* virulence remains to be elucidated. The role of the *K*. *rhinoscleromatis* CPS has never been tested *in vivo* since, currently, there are no *K*. *rhinoscleromatis* CPS mutants available.

As CPS is a prominent factor in other bacteria, here we explored the possibility that *K*. *rhinoscleromatis* CPS is implicated in the peculiar pathophysiological aspects of rhinoscleroma. We have previously established an intranasal mouse model of *K*. *rhinoscleromatis* infection recapitulating the formation of Mikulicz cells, the major histological feature of the disease [9]. In this work, we successfully constructed a *K*. *rhinoscleromatis* CPS mutant strain, representing the first report of the use of genetic tools in *K*. *rhinoscleromatis*. Further, using our mouse model, we compared the host responses to wild-type and *K*. *rhinoscleromatis* CPS mutant infections by examining cytokine production and pulmonary histology. We report that the *K*. *rhinoscleromatis* CPS mutant is attenuated *in vivo* but also that Mikulicz cells are observed upon infection with high dose of *K*. *rhinoscleromatis* CPS mutant. Our data indicate that capsule is a virulence factor of *K*. *rhinoscleromatis* but is not involved in the specific appearance of Mikulicz cells.

## Materials and methods

### Ethics statement

All protocols involving animal experiments were carried out in accordance with the ethical guidelines of Pasteur Institute, Paris and approved by the Comité d'Ethique de l'Institut Pasteur (CETEA) (comité d'éthique en expérimentation animale n°89) under the protocol license number: 2013–0031. All mice had free access to food and water and were under controlled light/dark cycle, temperature and humidity. Animals were handled with regard for pain alleviation of suffering. Animals were anesthetized using ketamine and xylazine, and euthanized with CO_2_.

### Bacterial strains, plasmids and media

Bacterial strains and plasmids used in this study are listed in Table 1. The *K*. *pneumoniae* subsp. *rhinoscleromatis* SB3432 strain (KR WT) was isolated in 2004 at the Avicenne hospital, Bobigny, France, from a biopsy of the left nasal cavity of an 11-years old patient diagnosed with rhinoscleroma. The *K*. *pneumoniae* subsp. *pneumoniae* Kp52145 strain is a previously described clinical isolate (serotype O1:K2) [25]. The *Escherichia coli* strains used in the cloning experiments were DH5α λ*pir* (Invitrogen) and ß2163, kind gift from Didier Mazel (Institut Pasteur, France). pGEM-T (Promega) is TA cloning vector used for cloning PCR products. pDS132 was a kind gift from Dominique Schneider (Université Joseph Fourier, France). A kanamycin cassette was PCR amplified from the plasmid pKD4 [26] and recombineering plasmid pSIM6 expressing Red system was used to create mutant in Kp52145 [27]. The plasmid pAT881 carrying the *luxABCDE* operon was used to make bioluminescent strains [28].

**Table 1:** Bacterial strains and plasmids used in this study.

**Strain**	**Description**	**Reference or source**
***E*. *coli***		
DH5α λpir	F- Δ*lac169 rpoS(Am) robA1 creC510 hsdR514 endA recA1 uidA(*Δ*MluI)*::*pir*	Invitrogen
β2163	(F−) RP4-2-Tc::Mu Δ*dapA*::(*erm-pir*) [Km^R^ Em^R^]	[32]
***K*. *rhinoscleromatis***		
KR WT (strain SB3432)	Wild-type; K3 serotype	[6]
KR-*lux*	Transformant from KR WT harbouring pAT881	This study
KR *cps*^-^	Capsule mutant harbouring pDS132	This study
KR *cps*^—^*lux*	Capsule mutant harbouring pAT881	This study
***K*. *pneumoniae***		
Kp52145	Wild-type; K2 serotype	[25]
Kp52-pSIM6	Kp52145 wild-type harbouring pSIM6	This study
Kp52Δ*wzc*	Capsule mutant; the *wzc* gene was inactivated	This study
**Plasmid**	**Description**	**Reference or source**
pGEM-T	Cloning vector	Promega
pDS132	Derived from pCVD442 (*R6K ori*, *mobRP4*, *bla*, *sacB*), without IS*l* sequences, *bla* gene replaced by *cat* gene (Cm^R^)	[33]
pKD4	Template plasmid carrying kanamycin resistance gene flanked by FRT sites	[26]
pSIM6	*red* genes expression vector (Amp^R^); low copy number (pSC101 replication origin)	[27]
pAT881	pGB2Ω*P*_*ami*_*luxABCDE*	[28]
pAM1	pGEM-T vector with the *wzb-kana-wbaP* fragment inserted in SacI site	This study
pAM2	pDS132 vector with the *wzb-kana-wbaP* fragment inserted in SacI site	This study

Bacteria were grown in Lysogeny Broth (LB) medium at 37°C with shaking. When appropriate, antibiotics were added at the following concentrations: ampicillin (Amp) 100 μg/ml; chloramphenicol (Cm) 30 μg/ml; kanamycin (Kan) 50 μg/ml. When necessary, DAP was supplemented to a final concentration of 0,3 mM. For selection against *sacB*, LB medium was supplemented with sucrose to a final concentration of 5% (wt/vol).

Inocula were prepared from overnight bacterial cultures grown on a loan on LB plates at 37°C resuspended in physiological saline.

### Construction of a *cps* mutant in *K*. *rhinoscleromatis* and in *K*. *pneumoniae*

Capsule *K*. *rhinoscleromatis* mutant (KR *cps*^*-*^) was obtained by insertion of the plasmid pAM2 in the *wzc* gene. Briefly, a kanamycin cassette flanked by 1kb of upstream (*wzb*) and 1 kb of downstream (*wbaP*) sequences of *wzc* using a three-step PCR method [29] was cloned into pGEM-T and then subcloned into pDS132 suicide vector. The resulting plasmid was introduced in the *E*. *coli* ß2163 donor strain (DAP^-^) and the recombinant strain was used for conjugation with *K*. *rhinoscleromatis*. KR *cps*^*-*^ mutants were selected onto Kan/DAP^-^ plates.

*K*. *pneumoniae* 52Δ*wzc* (Kp52Δ*wzc*) was generated using the λ RED recombination technique [26]. Briefly, a kanamycin cassette was amplified by PCR from the pKD4 plasmid using primers Kp52WzcUpKan (5’-ATCAGTGTTCAAACTTATTGAGCAATCTGCACTGTTATGGGCTGAGAAATTAAAAGCTTAGAAATTCAGGAAATAATGCATGATTGAACAAGATGGATTG -3’) and Kp52WzcDownKan (5’- CGATATGGATGACGTTCATTATTATCCTTTTATTATATATTTTAAAAAAGGGGATTCTTCGTCCCCTTCTTGAGTAACTCAGAAGAACTCGTCAAGAAGG -3’). The PCR product was purified onto a column, digested with *DpnI*, repurified and electroporated into *K*. *pneumoniae* carrying pSIM6, which encodes the λ RED recombinase. Kan-resistant clones were screened for successful genomic replacement of the entire *wzc*. Deletion of *wzc* on the *K*. *pneumoniae* 52145 chromosome was confirmed by PCR and sequencing.

### Infection of mice, determination of CFUs and LD50

Female BALB/cJ mice were purchased from Janvier (Le Genest-Saint-Isle, France).

Inocula of WT and mutant bacteria used in this study are 2.10^7^ bacteria for KR WT, 2.10^7^, 4.10^8^ and 10^9^ for KR *cps*^*-*^ and 10^9^ for Kp52Δ*wzc*. When appropriate, similar inocula of the respective bioluminescent strains were used.

Bacterial counts were determined as colony forming units (CFU) by plating serial dilutions of lung homogenates in 3 ml ice-cold PBS supplemented with 0,5% Triton X-100 and EDTA-free protease inhibitors (Fisher Scientific).

For survival studies, mice received either 2.10^7^ KR WT or 2.10^7^, 4.10^8^, 10^9^ KR *cps*^*-*^ by the intranasal route. Following infection, animals were returned to standard housing and observed for 14 days. A census of survivors was taken daily.

### Bioluminescence imaging

In order to maintain the plasmid conferring luciferase expression, mice were injected intraperitoneally twice daily from 1 day post-infection with 20 mg/kg spectinomycin (Spectam). Following isoflurane anesthesia, bioluminescence imaging was performed using an IVIS Spectrum (Perkin Elmer). Analysis and quantification of bioluminescence were done using Living Image (Perkin Elmer).

### Histology and Fluorescence In situ Hybridization (FISH)

At 96h post-infection lungs were inflated with 4% PFA and fixed overnight at 4°C. Paraffin-embedded tissue blocks were cut into 7 μm sections and stained with hematoxylin-eosin (HE). Images were acquired with the AxioScan.Z1 (Zeiss) using the Zeiss Zen2 software.

FISH staining was performed as follows. Paraffin lung sections were deparaffinized, rehydrated in PBS and covered with a solution of lysozyme at 10 mg/ml in PBS during 30 min at 37°C. Slides were then washed twice in PBS, preincubated 30 min at 42°C in hybridization buffer (20 mM Tris-HCl [pH 8], 0.9 M NaCl, 0.01% SDS, 30% formamide) and incubated overnight at 55°C in hybridization buffer containing 50 nM of the pan-bacteria probe Eub338-Alexa555 5′-GCTGCCTCCCGTAGGAGT-3 [30]. After washing in 1X SSC (1 SSC is 0.15 M NaCl plus 0.015 M sodium citrate), slides were covered for 1 min with DAPI to visualize the nuclei, washed in PBS and mounted in Prolong Gold reagent. Images were acquired on an upright fluorescence microscope equipped with the Apotome technology (Zeiss AxioImager with Apotome2, Carl Zeiss Jena).

### Images analysis

The number of Mikulicz cells was estimated from HE stained sections by manually segmenting region containing high number of Mikulicz cells in Zen Blue software (Zeiss). Regions containing Mikulicz cells within dense infiltrate of inflammatory cells were not included. Mikulicz cells-containing region was quantified as % area of the total lung area.

The number of bacteria present in the tissue section was quantified from fluorescence images using the Fiji plugin TrackMate [31]. Bacteria were defined as spots of 1.5 μm after Laplace Gaussian fitting.

### Capsule quantification

Capsule was quantified as the concentration of uronic acid in the samples from a standard curve of D-glucuronic acid as described by Favre-Bonte et al [14]. The uronic acid content was expressed in nanograms per 10^6^ CFU.

### Quantification of cytokines by ELISA

At various time post-infection the five pulmonary lobes were removed and collected in ceramic-beads containing tubes (Precellys lysing kit CK28) with 2,5 ml of ice cold PBS supplemented with 0,5% Triton X-100 and EDTA-free protease inhibitors (Fisher Scientific). Samples were then crushed using the Precellys homogenizer with the following program: 3 cycles of 15 sec at 5.000 × g with 10 sec pause. Twenty microliters were removed to determine the number of CFU/lung. After adding 10 μl of Pen/Strep (100X, Sigma), samples were centrifuged at 300 × g for 10 min and left on ice for 30 min. The supernatants were frozen rapidly in dry-ice ethanol bath and stored at -80°C. The following cytokines were measured: IL1ß, IL-10, IL-17, TNFα (Duoset, all from R&D Systems). Assays were performed according to the manufacturer’s instructions.

### Statistical analysis

Correlation between bioluminescence signal and CFU number was analyzed by Pearson correlation using GraphPad Prism 5.

## Results

### Genotypic and phenotypic characterization of the *K*. *rhinoscleromatis* capsule mutant strain

**Fig 1:**
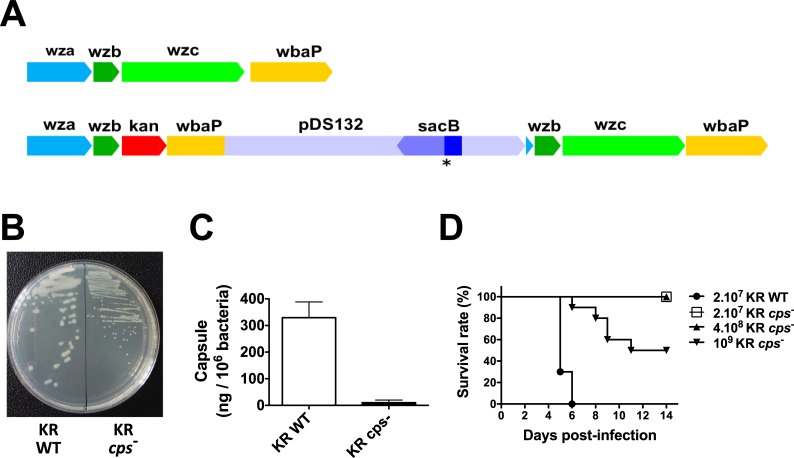
Characterization of the *K*. *rhinoscleromatis* capsule mutant strain (KR *cps*^*-*^). (A) Schematic representation of the wild-type *K*. *rhinoscleromatis* (top) and KR *cps*^*-*^ (bottom) capsule export region of the capsule locus. The insertion of the suicide plasmid pDS132 occurred at the level of the *wzb* gene. The asterisk indicates the base deletion in the *sacB* gene leading to aberrant protein production. (B) Morphology of the colonies of KR WT and KR *cps*^*-*^ strains after overnight culture on LB agar plates. (C) Quantification of capsule expressed as amount of uronic acid / 10^6^ bacteria in KR WT and KR *cps*^*-*^. (D) Mice survival after pulmonary infection with KR WT or KR *cps*^*-*^ strains. BALB/c mice were infected with 2.10^7^ KR WT, 2.10^7^ KR *cps*^*-*^, 4.10^8^ KR *cps*^*-*^ or 10^9^ KR *cps*^*-*^. Survival was followed overtime. Data are representative of 10 mice per group from two independent experiments.

To investigate the role of capsule in *K*. *rhinoscleromatis* virulence, we constructed a KR capsule mutant (KR *cps*^*-*^) from the *K*. *rhinoscleromatis* wild-type strain SB3432 (KR WT) by insertion of a suicide plasmid. It has been shown that inactivation of *wzc* gene, whose product is involved in capsular polysaccharide export machinery, leads to a capsule-minus phenotype in *K*. *pneumoniae* [13]. We decided thus to mutate the capsular operon in SB3432 by replacing the *wzc* gene by a kanamycin cassette by using the suicide plasmid pAM2. Although this suicide plasmid can be normally excised following double crossover using *sacB* counter-selection, we did not manage to obtain the desired gene replacement, possibly because KR does not grow on media without salt which is required for *sacB* counter-selection. Nevertheless, sequencing of KR *cps*^-^ confirmed the integration of the suicide plasmid in the *wzb* gene leading to a polar effect and one base deletion in the *sacB* gene leading to the production of a truncated SacB protein, hence explaining the selection of this mutant during the counter-selection step. A schematic representation of the wild-type KR and the capsule mutant KR *cps*^-^ capsule export portion of *cps* operon is shown in Fig 1A. As expected, colonies of KR *cps*^*-*^ did not show the slimy and mucoid phenotype characteristic of surface polysaccharide-producing KR colonies (Fig 1B). We also quantified the amount of capsule produced and observed a drastic reduction from 329±59 to 10±9 ng uronic acid / 10^6^ bacteria for KR WT and KR *cps*^*-*^ respectively. Altogether, these results indicated that this KR mutant is an effective capsule mutant.

Capsule is a well-characterized virulence factor of *K*. *pneumoniae*. *K*. *pneumoniae* capsule mutants are avirulent and they are not able to cause pneumonia or urinary tract infections [13,19,34]. We sought to analyze whether the KR *cps*^*-*^ strain was attenuated *in vivo*. Anticipating that at identical inoculum of KR WT and KR *cps*^*-*^ this would be the case, we wondered whether we could recapitulate part of the disease by increasing the infectious dose of the KR *cps*^*-*^. BALB/c mice were thus infected intranasally with 2.10^7^ KR WT or 2.10^7^, 4.10^8^ or 10^9^ KR *cps*^*-*^ and survival was monitored over 14 days (Fig 1C). While all mice infected with 2.10^7^ bacteria of KR WT strain succumbed within 6 days post-infection, mice infected with 2.10^7^ or 4.10^8^ KR *cps*^*-*^ bacteria recovered from the infection and survived. However, a 50% death rate was observed with the highest dose of 10^9^ KR *cps*^*-*^. Altogether, these findings show that the KR *cps*^*-*^strain is attenuated *in vivo*, confirming the crucial role of capsule in KR virulence.

### The capsule is a virulence factor of *K*. *rhinoscleromatis*

**Fig 2:**
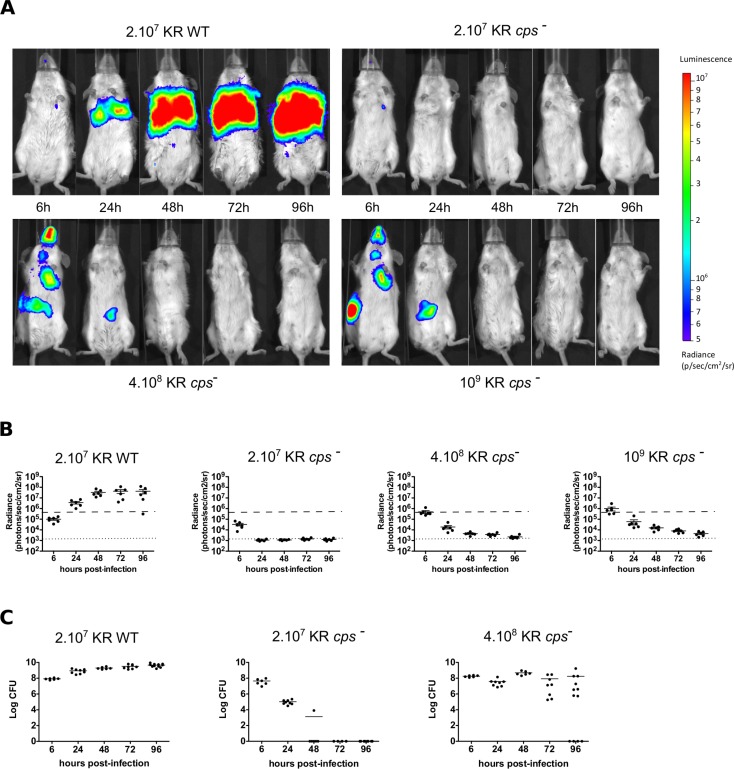
Bioluminescence imaging and bacterial loads quantification in mice infected by wild-type *K*. *rhinoscleromatis* and KR *cps*^*-*^. (A) BALB/c mice were infected with bioluminescent 2.10^7^ KR WT, 2.10^7^ KR *cps*^*-*^, 4.10^8^ KR *cps*^*-*^, and 10^9^ KR *cps*^*-*^. The bioluminescent signal in lungs was measured 6, 24, 48, 72, and 96 hours post-infection using the IVIS Imaging System. All images are shown using the same bioluminescence signal intensity scale (in photons/sec/cm^2^/sr). (B) Quantification of bioluminescent signal detected from six mice per group. Means are indicated as line. The dotted line indicates background level, and the dashed line shows the minimal signal shown in (A). (C) Bacterial load in lungs of mice infected with 2.10^7^ KR WT (left), 2.10^7^ KR *cps*^*-*^ (centre) or 4.10^8^ KR *cps*^*-*^ (right). Data for bacterial loads are shown as log CFU per organ from 6 to 12 mice from two to five independent experiments. Means are indicated as line.

In order to compare KR WT and KR *cps*^*-*^ infections, we tested the capacity of bioluminescent bacteria to colonize the lungs after intranasal instillation. Mice were infected with either 2.10^7^ bioluminescent KR WT or 2.10^7^, 4.10^8^ or 10^9^ bioluminescent KR *cps*^*-*^, and bioluminescence imaging was performed and quantified 6, 24, 48, 72, 96 hours post-infection (Fig 2A and 2B). Mice infected with bioluminescent KR WT showed a gradual increase in lungs bioluminescence with a 430 fold signal increase at 4 days post-infection as compared after 6 hours. On the other hand, the bioluminescence signal started to decrease from 6 hours post-infection with 2.10^7^ KR *cps*^*-*^ and reached background level at day 1. A similar but less pronounced decrease was observed in mice infected with 4.10^8^ or 10^9^ KR *cps*^*-*^ indicating a higher persistence of the mutant bacteria in the lungs. Moreover, because of a more viscous inoculum at high infection doses leading to difficulties to achieve proper intranasal infection, some mice swallowed part of the inoculum and showed a bioluminescent signal in the gut that disappeared in most of the animals at day 4, indicating that the bacteria transited in the gut before being eliminated. To correlate the bioluminescent signal with the bacterial load, we quantified the number of CFU in the lungs after bioluminescence imaging 96 hours post-infection. After subtraction of the background signal, we observed a significant correlation between bioluminescence and CFU in mice infected with 2.10^7^ KR WT and 4.10^8^ or 10^9^ KR *cps*^*-*^ (S1 Fig), allowing a good estimate of CFU greater than 5.10^5^ bacteria in the lungs from the bioluminescence signal.

We also directly monitored the lungs bacterial load during the same time course in mice infected with inocula of 2.10^7^ KR WT or 2.10^7^, 4.10^8^ KR *cps*^*-*^ (Fig 2C). While the number of bacteria in mice infected with 2.10^7^ KR WT gradually increased from 4.10^7^ bacteria per lungs 6 hours post-infection to reach 4.10^9^ bacteria at 96 hours, the number of bacteria in animals infected with the same inoculum of KR *cps*^*-*^ decreased gradually until the bacteria were being completely cleared from the organ in 72 hours. However, lungs from mice infected with a higher inoculum of 4.10^8^ KR *cps*^*-*^ presented a still significant amount of bacteria in the organ 4 days post-infection, providing a more relevant comparison to the wild-type infection. By 96 hours after infection with 4.10^8^ KR *cps*^*-*^, 33% of mice successfully cleared the infection while the others were still being colonized and had between 5.10^5^ and 10^9^ bacteria in their lungs. These results indicated that the KR *cps*^*-*^ mutant is strongly attenuated but that at a higher inoculum, after a certain threshold, KR *cps*^*-*^ is able to persist and proliferate within the host.

### The formation of Mikulicz cells in *K*. *rhinoscleromatis* is not capsule-dependent

**Fig 3:**
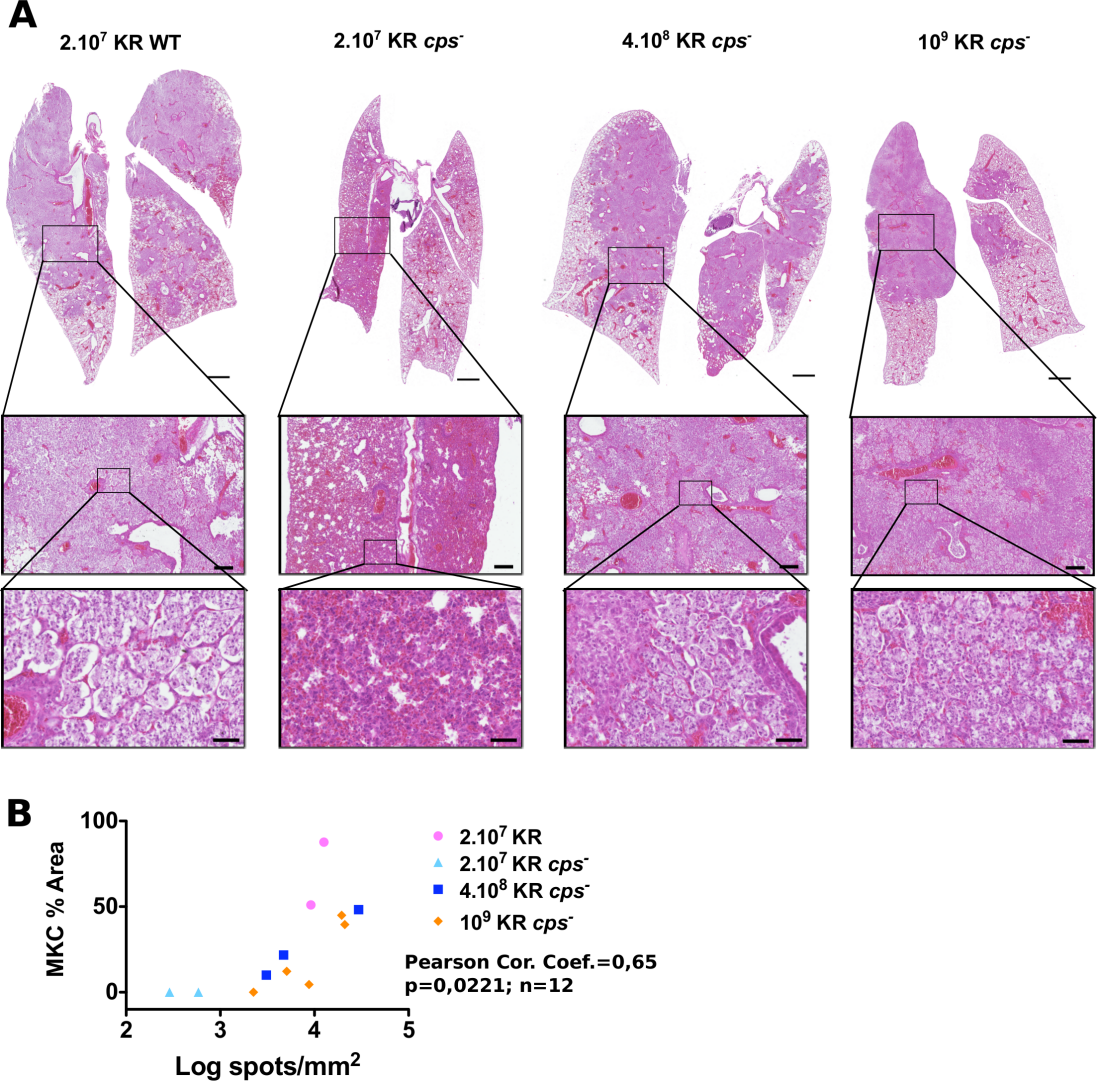
Histology of BALB/c lungs infected by wild-type *K*. *rhinoscleromatis* and KR *cps*^*-*^. (A) Lungs of mice infected with 2.10^7^ KR WT, 2.10^7^ KR *cps*^*-*^, 4.10^8^ KR *cps*^*-*^, and 10^9^ KR *cps*^*-*^ were resected 4 days post-infection and examined by histology. Lungs infected with 2.10^7^ KR WT (left) presented the classical pattern characterized by many alveoli, with an intact epithelial layer, filled with Mikulicz cells. On the contrary, lungs infected with 2.10^7^ KR *cps*^*-*^ (middle left) showed dense inflammatory infiltrate containing numerous polymorphonuclear cells and absence of Mikulicz cells. Lungs of mice infected with 4.10^8^ KR *cps*^*-*^ (middle right) or 10^9^ KR (right) showed the presence of Mikulicz cells similarly to wild-type infection. Insets show magnification of representative zones of the lungs. Scale bars are 1 mm (top), 200 μm (middle row) and 50 μm (bottom). Images are representative of 4 (KR WT), 4 (2.10^7^ KR *cps*^*-*^), 4 (4.10^8^ KR *cps*^*-*^) or 5 (10^9^ KR *cps*^*-*^) mice from 2 to 3 independent experiments. (B) Correlation between the area covered by Mikulicz cells and number of bacteria spots in lungs sections of mice at 96 hours after infection with 2.10^7^ KR WT, 2.10^7^ KR *cps*^*-*^, 4.10^8^ KR *cps*^*-*^, 10^9^ KR *cps*^*-*^.

To examine the pathology induced by KR *cps*^*-*^, lungs of mice were also examined histologically at 4 days post-infection (Fig 3A). Animals infected by 2.10^7^ KR WT presented the classical extensive but moderately destructive inflammation of the lungs characterized by the recruitment and formation of large Mikulicz cells filling alveoli. By contrast, mice infected with 2.10^7^ KR *cps*^*-*^ showed localized dense inflammatory lesions with signs of hemorrhages and recruitment of monocytic and polymorphonuclear cells. No classical Mikulicz cells could be observed. This phenotype reflects the inflammatory response that was required to eradicate the bacteria. Interestingly, when mice were challenged with 4.10^8^ or 10^9^ KR *cps*^*-*^, many alveoli were filled almost exclusively with Mikulicz cells, similarly to what is observed with KR WT, although the alveolar lining was more often disrupted. Regions with dense and localized inflammatory regions, characterized by infiltration of numerous polymorphonuclear cells, were also observed (Fig 3A, highlighted zone). Of note, all mice infected with 4.10^8^ or 10^9^ KR *cps*^*-*^ out of 9 examined histologically presented Mikulicz cells. Altogether, these observations suggested that while capsule is a virulence factor in KR, it is not required to induce the formation of Mikulicz cells in KR pathogenesis.

As mice infected with the KR *cps*^*-*^ strain showed variations in the intensity of the Mikulicz cells infiltrate observed by histology, we wondered whether this variation was correlated to the bacterial burden. Because we cannot directly quantify total CFU and perform an histological analysis on the same sample, we estimated the number of bacteria by fluorescence in situ hybridization and quantified the Mikulicz cells infiltrate by manually segmenting regions containing highly visible Mikulicz cells on adjacent lungs sections (S2 Fig). Mice infected with KR *cps*^*-*^ showed different number of bacteria spots and extend of Mikulicz cells infiltrate in the lung section (Fig 3B). Both parameters were significantly correlated, suggesting that the local bacterial load drives the intensity of recruitment of Mikulicz cells.

### IL-10 production is capsule-independent

**Fig 4:**
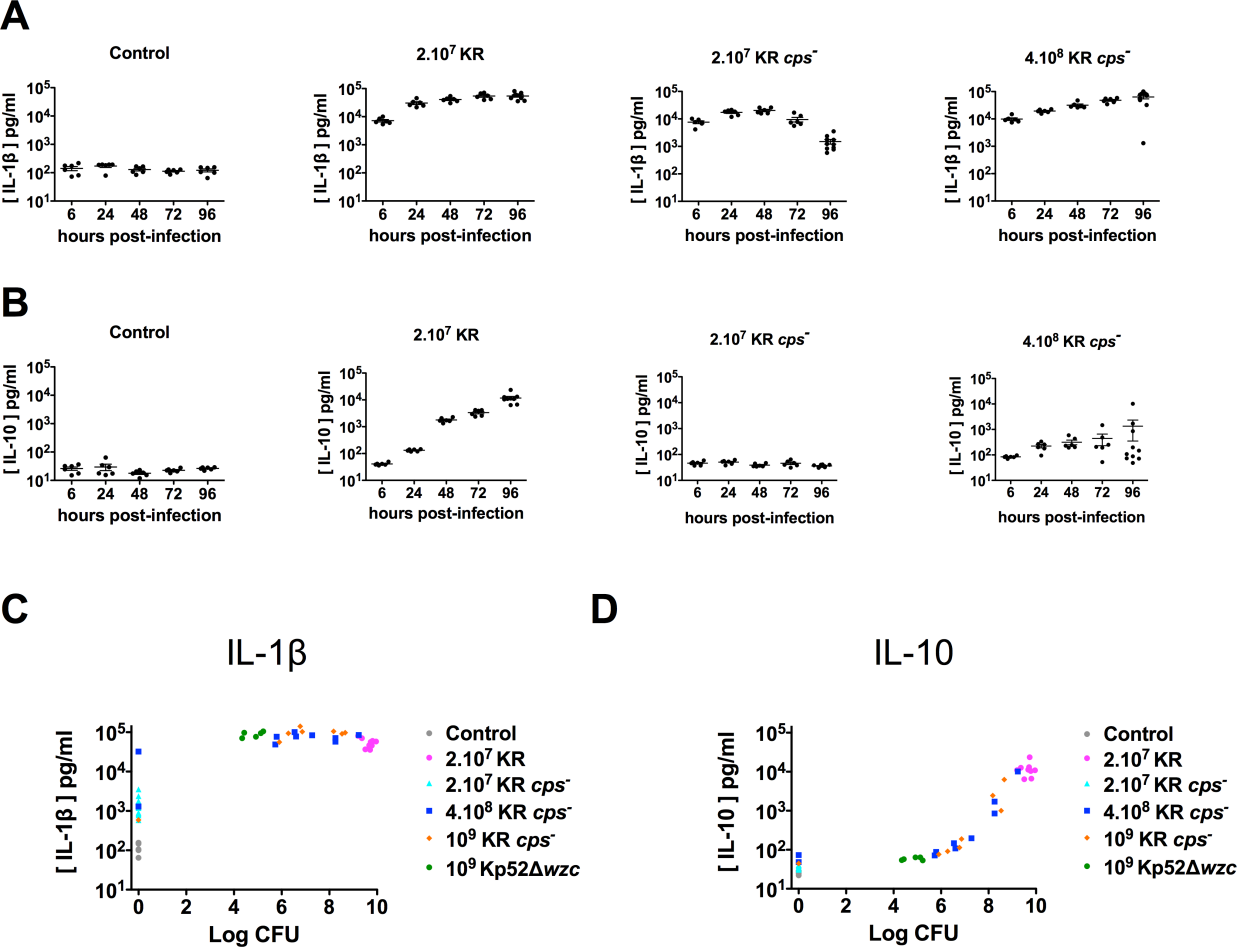
Production of IL-1β and IL-10 in lungs of BALB/c mice infected by wild-type *K*. *rhinoscleromatis*, KR *cps*^*-*^, Kp52Δ*wzc* or Kp52145. BALB/c mice were infected with 2.10^7^ KR WT, 2.10^7^ KR *cps*^*-*^ or 4.10^8^ KR *cps*^*-*^ or saline-injected for 6, 24, 48, 72 or 96 hours. Lungs were then homogenized and the pro-inflammatory IL-1β (A) and the anti-inflammatory IL-10 (B) cytokines were measured by ELISA. Data are mean from 6 to 10 mice from three independent experiments. Correlation between production of IL-1β (C) or IL-10 (D) and the amount of bacteria recovered from lungs of mice at 96 hours after injection with saline (control) or infection with 2.10^7^ KR WT, 2.10^7^ KR *cps*^*-*^, 4.10^8^ KR *cps*^*-*^, 10^9^ KR *cps*^*-*^, 10^9^ Kp52Δ*wzc* or 2.10^4^ Kp52145.

Cytokines are key mediators of immune responses and the anti-inflammatory cytokine IL-10 has been shown to be highly produced after *K*. *rhinoscleromatis* infection and to play a crucial role in the establishment of a proper environment leading to Mikulicz cells maturation [9]. Therefore, we characterized the production of some major cytokines in mouse lung extracts upon KR *cps*^*-*^ infection. When BALB/c mice were infected with 2.10^7^ KR WT or 4.10^8^ KR *cps*^-^, the pro-inflammatory cytokines IL-1β, IL-17 and TNF-α were produced in high amounts from 6 hours post-infection onwards (Fig 4A and S3 Fig). However, although produced in similar amounts at the beginning of the infection in mice infected with 2.10^7^ KR *cps*^*-*^, the level of these cytokines diminished overtime because bacteria were progressively cleared from the organ. As previously shown, the anti-inflammatory cytokine IL-10 was highly produced upon infection with 2.10^7^ KR WT but not in mice infected with 2.10^7^ KR *cps*^*-*^. IL-10 was also produced in mice infected with 4.10^8^ KR *cps*^-^, but to a lower extend and in more variable manner as compared to KR WT (Fig 4B). These observations indicate that a high inoculum of KR *cps*^*-*^ allows recapitulating a high production of IL-10, thereby suggesting that capsule does not have a direct role in IL-10 production upon KR infection.

Because we observed a high variability in the production of IL-10 in mice infected with 4.10^8^ KR *cps*^*-*^, we wondered whether it was correlated with the burden of the infection. We thus compared the production of IL-1β, IL-17, TNF-α and IL-10 to the number of CFU in the lungs at 96 hours post-infection for each animal. While a high production of IL-1β (> 3.10^4^ pg/ml) is indicative of the presence of bacteria in the lungs (mainly ranging from 10^5^−10^9^ bacteria), IL-1β is expressed at intermediate levels (500–3.000 pg/ml) when mice managed to clear the infection (Fig 4C). Similar observation was made for IL-17 and TNF-α (S4 Fig). On the other hand, this is different for IL-10 (Fig 4D). A first group of mice mildly colonized (between 5.10^5^ and 2.10^7^ CFU) showed intermediate level of IL-10 (between 70 and 200 pg/ml) while a second group of mice that were unable to control the infection (> 2.10^7^ CFU) were characterized by an intense production of IL-10 (> 10^3^ pg/ml) suggesting that KR is able to induce an intense production of IL-10 only above a certain threshold of KR bacteria in the lungs.

**Fig 5:**
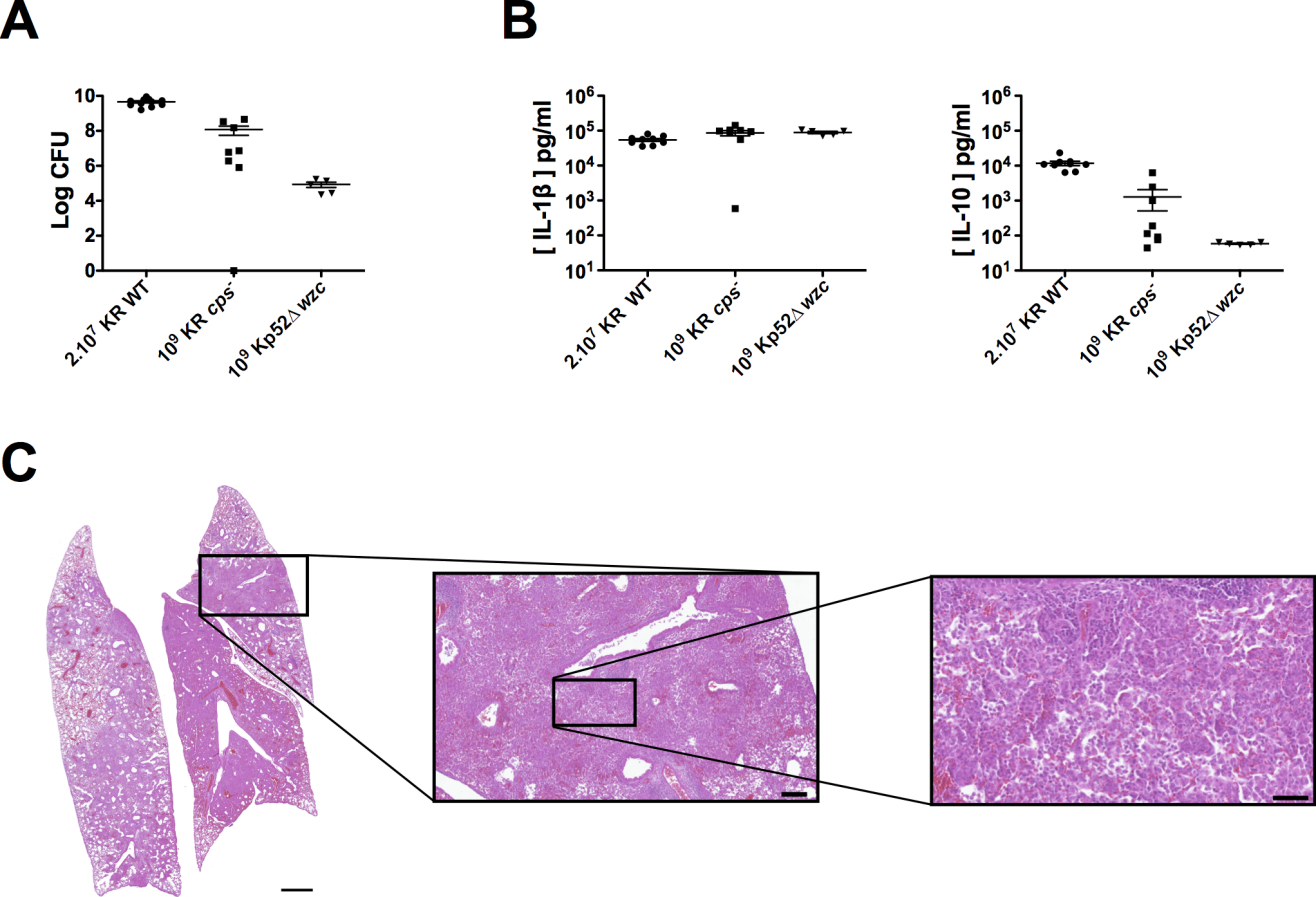
The capsule mutant Kp52Δ*wzc* is not able to induce the formation of Mikulicz cells. (A) Bacterial load in lungs of BALB/c mice after infection with 2.10^7^ KR WT, 10^9^ KR *cps*^*-*^ or 10^9^ Kp52Δ*wzc*. Data show CFU in whole lungs after 96 hours post-infection from 5 to 11 mice from one to five independent experiments. Means are indicated as line. (B) Production of IL-1β and IL-10 in the lungs of BALB/c mice infected with 2.10^7^ KR WT, 10^9^ KR *cps*^*-*^ or 10^9^ Kp52Δ*wzc*. Cytokines were measured 96 hours post-infection by ELISA. Data are mean from 5 to 9 mice from two independent experiments. (C) Histology, representative example of lung from mice infected with 10^9^ Kp52Δ*wzc*. Zones of dense inflammation can be observed with absence of Mikulicz cells. Scale bars are 1 mm (left), 200 μm (middle) and 50 μm (right).

To establish that the occurrence of Mikulicz cells observed with 4.10^8^ and 10^9^ KR *cps*^*-*^ was not due to the higher inoculum of KR *cps*^*-*^ as compared to KR, we measured bacterial loads and cytokines expression in animals inoculated with the same high inoculum (10^9^ bacteria) of Kp52Δ*wzc* 4 days post-instillation. The Kp52Δ*wzc* strain is a similar capsule mutant from *K*. *pneumoniae* strain Kp52145 obtained after deletion of the *wzc* gene showing a drastic reduction of capsule expression from 256±22 ng uronic acid / 10^6^ bacteria for Kp52145 to 34±13 ng uronic acid / 10^6^ bacteria (S5 Fig). We observed that the bacterial load of mice infected with 10^9^ Kp52Δ*wzc* was around 10^5^ bacteria per organ and was lower than the bacterial load of mice infected with 10^9^ KR *cps*^*-*^ indicating that a *wzc* mutant in Kp52145 is less virulent that its counterpart in KR (Fig 5A).

We then measured the cytokines levels in lungs of infected animals (Fig 5B and S6 Fig). Pro-inflammatory cytokines IL-1β, IL-17 and TNF-α were expressed in similar amounts in mice infected with Kp52Δ*wzc* or KR *cps*^*-*^. However, IL-10 was expressed at low level after 10^9^ Kp52Δ*wzc* infection (53–63 pg/ml), contrasting the higher amount observed after 10^9^ KR *cps*^*-*^ infection in some mice. By histology, we observed an intense and dense inflammation characterized by a strong recruitment of monocytes and polymorphonuclear cells and an absence of Mikulicz cells formation (Fig 5C). Altogether, and combined with the histological data, these observations suggest that when present in high concentration in the lungs from 3 days of infection without being lethal, KR or its capsule mutant are able to induce the recruitment and maturation of Mikulicz cells and drive a strong production of IL-10.

## Discussion

The diversity of capsule types in *Klebsiella pneumoniae* species is strikingly very large, as 134 different capsule loci have been identified up to now [21]. This tends to indicate that *K*. *pneumoniae* species is under strong selection pressure to diversify its capsule. However, and strikingly, all *K*. *rhinoscleromatis* strains isolated so far are of the KL3 (K3) serotype despite having been isolated from diverse geographical locations [6]. Because of this homogeneity, we speculated that this specific K3 serotype could be an important factor driving the rhinoscleroma disease. By creating a capsule mutant in *K*. *rhinoscleromatis*, we showed that if capsule is an important virulence factor for this species, it is not necessary to induce the formation of Mikulicz cells, the hallmark of rhinoscleroma, as these cells have been observed when using high inocula of this mutant.

The saccharide composition of the capsule has been linked to some extent to *K*. *pneumoniae* virulence. K1 and K2 serotypes have been suggested to be major determinants in liver abscess-causing *K*. *pneumoniae* [35,36]. Strains from other serotypes, including K5, K16, K20, K54 and K57, have also been described as highly virulent [37]. In addition, switching the capsular serotype of a highly virulent K2 strain to a weakly virulent K21a strain has been shown to lead to a decrease in virulence in mouse and in survival in blood and to an increased binding to macrophages. Conversely, switching the capsule serotype of the K21a strain to virulent K2 resulted in an increased virulence in mouse and in survival in blood and to a lower binding to macrophages [38,39]. In addition, switching highly conserved genes of the capsule cluster involved in capsule export from K1 into K20 hypervirulent strain strongly reduced its bacterial virulence in mice while increasing its neutrophil phagocytosis and survival in macrophages, although it is still not known whether this is due to a change in capsule expression[40]. However, a recent pan-genomic analysis did not reveal any correlation between capsule serotype and strains responsible of invasive community-acquired infection but rather suggested that the presence of one or several siderophores explains bacterial virulence [41]. Thus the exact role of capsule composition in virulence still remains to be clearly determined.

Capsule plays an important role in immune cells evasion by preventing binding of complement and antibodies to the bacteria thereby decreasing opsono-phagocytosis and complement-mediated killing [10–13,42–45]. Moreover, *Klebsiella* capsule composition has been shown to influence the binding of the bacteria to macrophages. K3, K46 and K64 *K*. *pneumoniae* capsule are binding more to the mannose receptor, which is highly expressed on macrophages, than other serotypes, in a mannose-dependent manner, while other serotypes presented no binding [23]. A common feature of these three different serotypes is that they have two or three mannose residues in their repeated unit. Though, other K serotypes that present also two mannose residues did not show any binding to the mannose receptor, suggesting that binding of mannose-bearing capsule to the mannose receptor is influenced by other factors than its mannose composition. However, as all *K*. *rhinoscleromatis* strains are of the K3 serotype, and even though K3 capsule interacts with mannose receptor, our results obtained with a high infection dose of KR *cps*^*-*^ suggest that this step is not important in driving the development of Mikulicz cells.

Some results obtained with the high inocula of KR *cps*^*-*^ were heterogeneous: the bacterial load 4 days post-infection was spread over 4 logs, IL-10 levels in the lungs were quite variable and some mice showed some bacteria in the digestive tract by bioluminescence. This variability is a consequence of the use of higher inocula, which are thicker and more viscous than lower inocula used for the KR WT and of KR *cps*^*-*^ strains that are more fluid. As a consequence, part of the inoculum is swallowed by mice and passes into the digestive tract. This is also suggesting that above a certain threshold of *cps*^*-*^ bacteria delivered to the lungs, the animal cannot control the infection and the bacteria are able to multiply and maintain themselves in high number, although to a lower burden than WT bacteria. We wondered whether there was a correlation between the number of bacteria in the lungs and the level of IL-10 produced. Indeed we observed that IL-10 was produced in high amount when the bacterial load was high, raising the possibility that high IL-10 expression was the result of a high bacterial burden and not specific to *K*. *rhinoscleromatis*. To verify this one needs to compare IL-10 production upon similar bacterial burdens, greater than 10^8^ bacteria, at 4 days post-infection with different bacteria. We first thought to use a high dose of a *K*. *pneumoniae* mutant inactivated in the same gene as the KR *cps*^*-*^ strain, but showed that the bacterial load was lower (10^4^−10^5^ bacteria) than the lowest ones obtained with high KR *cps*^*-*^ (10^6^ to 10^8^ bacteria) and that IL-10 levels were also quite low. This showed that this Kp52Δ*wzc* mutant was actually more attenuated than KR *cps*^*-*^ and suggested that *K*. *rhinoscleromatis* is better adapted to surviving in lungs. Some virulent *K*. *pneumoniae* strains can cause intense and severe and acute pneumonia in mice with high burden. We had previously observed that a variable bacterial load can be achieved 3 and 5 days post-infection with a low dose of the virulent strain Kp52145 [9] and that about 30% of mice were presenting a high bacteria burden 3 and 5 days post-infection. By measuring CFU loads and cytokines in mice infected with Kp52145 we observed that mice that had a high bacterial load were producing IL-10 in amount similar to those that were less colonized. Comparable high bacterial burden were obtained with the widely used *K*. *pneumoniae* strain 43816 [18,19,46] and IL-10 was produced in similar low amounts 3 days post-infection [18]. Hence these observations indicate that the intense IL-10 production observed upon infection with KR WT or KR *cps*^*-*^ is specific of *K*. *rhinoscleromatis* and does not result from a global high bacterial load.

Moreover, all high dose KR *cps*^*—*^infected mice out of 9 observed by histology show the presence of Mikulicz cells in their lungs, although to various extent. We also observed that the density of the Mikulicz cells infiltrate is correlated to the number of bacteria. We also tried to see whether there was a similar correlation with the amount of IL-10 on a mouse to mouse basis, but were unable to detect directly this cytokine by immunohistochemistry. Nevertheless, the variation in the host response to KR *cps*^*-*^ infection is likely correlated to the amount of IL-10 produced: lower number of bacteria lead to fewer Mikulicz cells and low amounts of IL-10 whereas an intense IL-10 production is accompanied by high number of bacteria and Mikulicz cells and less destructive inflammation.

Recently, IL-10 has been shown to regulate metabolic processes in activated macrophages and thus control the inflammatory response. IL-10 impedes glycolysis and promotes oxidative phosphorylation maintaining mitochondrial fitness. This metabolic reprogramming of macrophages is controlled by IL-10 through inhibition of mechanistic target of rapamycin (mTOR) signaling pathway [47]. Interestingly, deregulation of mTOR signaling, such as prolonged mTORC1 activation, leads to metabolic changes, hyperproliferation of macrophages and granuloma formation, contributing to disease progression in human granulomatous sarcoidosis [48]. These mechanisms might be associated with formation of granulomas in rhinoscleroma, where Mikulicz cells could undergo similar metabolic remodeling mediated by IL-10.

The fact that the capsule is not required for Mikulicz cells recruitment and formation indicates that the factors responsible of this process are still unknown and remain to be identified. Current *in vivo* screening approaches, such as signature tagged mutagenesis, cannot be used as they identify mutants unable to grow in specific experimental conditions, but not those that are required for the expression of a particular phenotype, such as the appearance of Mikulicz cells. Therefore an *in vitro* screening assay has to be developed. However, *in vivo* phagocytosis assays can often be difficult to set up and standardize due to the high expression of capsule in *K pneumoniae* species and its strong anti-phagocytic effect. Our results show that capsule is not required for the formation of Mikulicz cells, opening the way to *in vitro* assays of Mikulicz cells formation and to *in vitro* screening of factors that are driving this maturation *in vivo*.

## Supporting information

S1 Fig Bioluminescence signal and CFU correlation. Bioluminescence signal after background subtraction was correlated with the CFU number for mice infected with 2.10^7^ KR WT, 4.10^8^ KR *cps*^*-*^ and 10^9^ KR *cps*^*-*^.
(EPS)

S2 Fig Quantification of bacteria spots by FISH. Bacteria were detected in lungs section by FISH using pan bacteria probe. Bacteria spots were quantified using the plugin Trackmate in Fiji. (A) shows a representative image of a lung section from a mouse infected with 2.10^7^ KR (top left) with the bacteria labelled in orange, a high resolution zoom showing individual bacterial spots (middle left) and the corresponding spots detected by TrackMate (middle right). A corresponding HE image with the manually segmented area containing Mikulicz cells is shown (top right). (B) Representative image from a mouse infected with 2.10^7^ KR *cps*^*-*^. Bottom images in A and B are 140 μm wide.
(EPS)

S3 Fig Production of IL-17 and TNF-α in lungs of BALB/c mice infected by wild-type *K*. *rhinoscleromatis* or KR *cps*^*-*^. BALB/c mice were infected with 2.10^7^ KR WT, 2.10^7^ KR *cps*^*-*^ or 4.10^8^ KR *cps*^*-*^ or saline-injected for 6, 24, 48, 72 or 96 hours. Lungs were homogenized and the pro-inflammatory cytokines IL-17 (A) and TNF-α (B) were measured by ELISA. Data are mean from 6 to 10 mice from three independent experiments.
(EPS)

S4 Fig Cytokines production and CFU correlation. Correlation between production of IL-17 (A) or TNF-α (B) and the amount of bacteria recovered from lungs of mice at 96 hours after injection with saline or infection with 2.10^7^ KR WT, 2.10^7^ KR *cps*^*-*^, 4.10^8^ KR *cps*^*-*^, 10^9^ KR *cps*^*-*^, 10^9^ Kp52Δ*wzc* or 2.10^4^ Kp52145.
(EPS)

S5 Fig Quantification of capsule in Kp52145 and mutant Kp52Δwzc. Data are expressed as ng of uronic acid / 10^6^ bacteria.
(EPS)

S6 Fig **Production of IL-17 (A) and TNF-α (B) in lungs of BALB/c mice infected by wild-type *K*. *rhinoscleromatis*, KR *cps***^***-***^ **or Kp52Δ*wzc*.** BALB/c mice were infected with 2.10^7^ KR WT, 10^9^ KR *cps*^*-*^ or 10^9^ Kp52Δ*wzc*. Cytokines were measured 96 hours post-infection by ELISA. Data are mean from 5 to 9 mice from two independent experiments.
(EPS)
